# Altered structural connectivity in white matter lesion-induced mild cognitive impairment: a probabilistic fiber tracking and graph theory analysis

**DOI:** 10.3389/fncir.2026.1852574

**Published:** 2026-06-19

**Authors:** An-Ming Hu, Yan-Ling Ma, Yue-Xiu Li, Qing-li Shi, Yu-Mei Zhang

**Affiliations:** 1 Department of Rehabilitation Medicine, Beijing Tiantan Hospital, Capital Medical University, Beijing, China; 2 Beijing Xiaotangshan Hospital, Beijing, China; 3 Beijing Pinggu Hospital, Beijing, China

**Keywords:** brain structural network, cognitive impairment, diffusion tensor imaging, probabilistic fiber tracking, white matter lesions

## Abstract

White matter lesions (WMLs) are a common cause of vascular cognitive impairment, yet the nature of whole-brain structural connectivity alterations in WML-related mild cognitive impairment (MCI) remains incompletely understood. In this study, an integrated approach combining whole-brain probabilistic fiber tracking with graph theory analysis was employed to investigate structural network changes in 33 patients with WML-related MCI and 37 healthy controls. The results revealed that, at the MCI stage, the brain’s structural network exhibited not only reduced connectivity in specific neural circuits–primarily fronto–basal ganglia pathways–but also widespread increased connectivity, particularly between the frontal lobe and other cortical regions. Group comparisons identified 50 significantly altered connections (42 increased, 8 decreased), predominantly involving cortico–cortical, cortico–deep gray matter, and deep gray matter–deep gray matter pathways. Despite these connection-level abnormalities, no significant differences were observed in global or nodal graph-theoretical metrics. Notably, eight of the altered connections showed significant correlations with Montreal Cognitive Assessment (MoCA) scores. These structural alterations were closely associated with cognitive and behavioral deficits, indicating that they might serve as exploratory imaging indicators for WML-induced cognitive impairment.

## 1 Introduction

White matter lesions (WMLs), also termed leukoaraiosis, are a hallmark of cerebral small vessel disease characterized by hyperintensities on T2-weighted and FLAIR (fluid-attenuated inversion recovery) MRI sequences. Pathologically, WMLs represent microvascular-induced white matter damage, with progressive degeneration linked to chronic hypoperfusion and blood-brain barrier dysfunction (Tuladhar et al., 2015; Chen et al., 2021). Mild cognitive impairment (MCI), a common clinical manifestation in WML patients, often emerges in early disease stages and progresses to dementia over time, highlighting the urgent need to elucidate its underlying neural mechanisms (Rost et al., 2022).

While functional MRI (fMRI) has been extensively used to investigate disrupted functional connectivity in WML-related cognitive impairment, structural network alterations remain relatively underexplored (Yueniwati et al., 2018; Ingwersen et al., 2024). Diffusion tensor imaging (DTI)-based white matter structural networks offer a complementary approach for quantifying whole-brain connectivity. Structural network abnormalities, such as altered connectivity probabilities, are closely associated with pathological conditions like microvascular damage and microbleeds, serving as sensitive biomarkers for WML progression. Moreover, DTI-based structural connectivity analysis provides a robust framework for assessing cerebral white matter microstructural integrity and network topology (Tuladhar et al., 2017).

Moreover, accumulating evidence further indicates that white matter lesions (WMLs) have a significant impact on brain structural connectivity. WMLs not only disrupt the physical integrity of specific white matter fiber tracts but also lead to dysregulation of whole-brain structural network topology (Busby et al., 2025; Petersen et al., 2024). For example, WML-related disconnection in association fibers, particularly the superior longitudinal fasciculus and frontal aslant tract, has been shown to be significantly correlated with lower MoCA scores and memory impairment (Busby et al., 2025). A large multicenter study further demonstrated that WML connectivity metrics derived from lesion network mapping significantly outperform traditional WML volume measurements in predicting cognitive performance across domains such as attention/executive function, processing speed, and verbal memory (Petersen et al., 2024). These findings suggest that WML-induced cognitive impairment arises not only from focal white matter damage but also from widespread alterations in structural brain networks.

White matter fiber tractography is a technique that uses DTI data to estimate the probability distribution of axonal orientations and thereby quantify the strength of structural connectivity between different brain regions (Petersen et al., 2024). Currently, two main approaches are widely employed: (1) deterministic tractography, which constructs well-defined white matter pathways by linking fiber segments within each voxel along a fixed direction (Chen et al., 2018); and (2) probabilistic tractography, which assumes that the fiber orientation within each voxel follows a probability density distribution. Starting from a seed point, this method performs multi-step sampling based on the local orientation probability distribution, ultimately generating multiple plausible pathways and computing a corresponding connection probability (Abos et al., 2019).

Structural connectivity probability can be interpreted as a quantitative metric of neural signal transmission capacity. It reflects the efficiency of neural information propagation within the brain network and serves as a key parameter for assessing structural connectome integrity (Battocchio et al., 2022). Compared with deterministic tractography, probabilistic tractography offers significant advantages in handling noise, effectively addresses the challenge of fiber crossings, and enables more accurate reconstruction of white matter pathways (Bucci et al., 2013).

The threshold-free network-based statistics (TFNBS) method enables sensitive detection of alterations in the organization and topology of white matter fiber bundles. Unlike traditional cluster-based approaches, TFNBS generates edge-wise statistical significance without requiring an *a priori*–defined hard threshold for cluster formation, thereby reducing bias and enhancing reproducibility (Baggio et al., 2018). Graph theory, meanwhile, provides a powerful mathematical framework for characterizing the architecture and dynamic reorganization of complex brain networks (Sporns, 2018). The integration of probabilistic fiber tracking with graph-theoretical analysis has recently been applied to uncover disruptions in network integration and segregation within the whole-brain structural connectome of patients with various neurological disorders (Inguanzo et al., 2021; Feng et al., 2022).

To our knowledge, few studies have investigated structural connectivity alterations in white matter lesions (WMLs) using an integrated approach that combines whole-brain probabilistic fiber tracking, graph theory analysis, and threshold-free network-based statistics (TFNBS). The present study aims to characterize large-scale changes in structural brain connectivity in patients with WML-related mild cognitive impairment (MCI) using this multimodal framework, thereby providing a neurobiological foundation for the early detection of cognitive decline.

## 2 Materials and methods

### 2.1 Ethical approval

This study was conducted in accordance with the Declaration of Helsinki. The research procedures were approved by the Ethics Committee of Beijing Tiantan Hospital Affiliated with Capital Medical University (Approval No.: KYSB2016.023). Formal written informed consent was obtained from all participating subjects.

### 2.2 Research subjects

This is a retrospective study. Patients with right-handed WMLs in the brain who underwent an MRI examination in Beijing Tiantan Hospital, from 2014 to 2018, were included. Two radiologists independently diagnosed and recruited patients with WMLs according to the European Neuroimaging Standards for Measuring and Reporting Vascular Changes in Neurodegeneration (Wardlaw et al., 2013). The diagnostic imaging physician was blinded to the clinical data of the patients.

According to the revised Fazekas scale, the inclusion criteria of patients with WMLs included (1) the presence of WMLs on MRI and (2) the age between 40 and 85 years old. Based on the Montreal Cognitive Assessment (MoCA) and Clinical Dementia Rating (CDR) scales, the patients with WMLs were diagnosed with vascular cognitive impairment with no dementia (VCIND) (Morris, 1993; Magierska et al., 2012). Healthy controls (HCs), aged 40–85 years, were recruited from the community. The exclusion criteria of WMLs and HCs included (1) patients with heart or renal failure, cancer, or other serious systemic diseases, (2) patients suffering from other nervous system diseases, such as epilepsy, traumatic brain injury, and multiple sclerosis, etc., (3) patients with depression and cognitive impairment caused by a primary neurological disease with certain unique features (such as Alzheimer’s disease, frontotemporal lobe deformation, Parkinson’s syndrome, etc.), (4) patients with non-vascular dementia, (5) patients with a psychiatric disorder or drug addiction, (6) patients with impaired consciousness or aphasia, (7) patients who are unable to cooperate with the cognitive function evaluations, and (8) patients who refuse or are unable to be examined via cranial MRI.

Finally, 33 WML-MCI patients and 37 healthy controls were included in the final statistical analysis.

### 2.3 Basic data collection

Basic patient data such as basic clinical and demographic information (age, gender, years of education, etc.) were collected.

### 2.4 Cognitive assessment

Cognitive functions were evaluated by trained neurologists in a quiet room without external interference. The CDR and MoCA were used for cognitive evaluation.

The MoCA was used to screen the overall cognitive function. It assessed 7 cognitive domains: visuospatial abilities, naming, attention, language, abstraction, memory, and orientation to time and place (with accuracy confirmation). For patients with cognitive impairment, CDR was used to assess the severity of the impairment. According to the diagnostic criteria of vascular dementia by the National Institute of Neurological Disorders and Stroke/Swiss Society for Neuroscience, the MRI findings of patients in the vascular cognitive impairment non-dementia group (WML-MCI group) were consistent with WMLs, and scored 0.5 points in the CDR and <26 points in the MoCA (Morris, 1993). According to the MoCA and CDR results, 33 patients were identified as having WMLs with VCIND. 37 volunteers with normal cognition and no WMLs were selected as the control group.

### 2.5 MRI data acquisition

All subjects underwent brain image acquisition at Beijing Tiantan Hospital using a 3.0T magnetic resonance imaging system manufactured by Siemens in Germany. The subjects lay on their back on the examination bed with their heads fixed. The subjects remained calm and awake, and did not engage in any brain activities, such as thinking, during the scan. DTI images were acquired using a single-shot, twice-refocused, diffusion-weighted echo planar imaging sequence with the following scan parameters: repetition time (TR) = 8000 ms; echo time (TE) = 96 ms; 64 diffusion-weighted directions with a b value of 1000 s/mm^2^ and11 images with a b value of 0 s/mm^2^; flip angle = 90°; field of view = 224 mm^2^; in-plane resolution = 1.75 mm × 1.75 mm voxels; and 54 contiguous 2 mm thick axial slices.

### 2.6 MRI data analysis

(1) DTI data pre-processing

The pre-processing of DTI data was performed using the PANDA software^1^. The pre-processing steps were as follows. (1) Format conversion: the dcm2nii tool in the MRIcron software was used to embed and convert DICOM format images to NIfTI format. (2) Estimation of the whole brain mask: the BET command was used to remove the skull and non-brain tissue parts to obtain the whole brain mask. (3) Construction of an image that included only the whole brain tissue to replace the original image, which greatly reduced the amount of data to be analyzed, without omitting any brain information, thereby reducing the memory requirement of the system and speeding up the subsequent analysis. (4) Eddy current and head movement correction: the b0 image was extracted, and each diffusion-weighted image was aligned to the b0 image using affine transformation to correct for distortions caused by eddy current and the motion artifacts caused by excessive head movements, while the diffusion gradient direction was adjusted accordingly; (5) The fractional anisotropy (FA) at the voxel level was calculated using the DTIFIT command, and the raw FA map was generated.

(2) Construction of brain structure networks

All probabilistic tractography analyses were performed using the default parameters of the PANDA toolbox, which calls FSL’s probtrackx2 algorithm. Specifically, 5000 streamlines were generated per seed voxel with a step length of 0.5 mm, a curvature threshold of 0.2, a minimum streamline length of 0.5 mm, and a maximum of 2000 steps per streamline. Tractography was restricted to white matter voxels defined by FA > 0.2, and tracking was terminated when streamlines exited the white matter mask or exceeded the curvature threshold. Connection probabilities were normalized row-wise, where the strength of connection between region i and region j was calculated as the number of streamlines from i to j divided by the total number of streamlines originating from region i.

i) Definition of network nodes: the brain was segmented into 90 brain regions using the automatic anatomical labeling (AAL) template as the nodes of the brain structural network. First, the individual B0 images were registered to the Montreal Neurological Institute 152 (MNI-ICBM152) template, and then the inverse transformation process was used to register the AAL template from the MNI space to the DTI space, so that the brain could be partitioned in an individual space to obtain the individual AAL template.
ii) Definition of edge (edges) of the network: using each brain region of the AAL as a node, the PANDA software was used to perform probabilistic fiber tracking (OPD type) to obtain a probability connection matrix of 90 × 90. To ensure the reliability of the node-node connections, a specific node-node connection was retained only if more than 50% of the subjects have a connection between the two nodes. Otherwise, the connection was considered false and set to 0.

Quality control steps: For each subject, we visually inspected the preprocessed results (including b0 images, FA maps, and brain extraction results). Subjects with excessive head motion (translation >3 mm or rotation >3°) or obvious image artifacts were excluded. Qualitative inspection of fiber tracking results was performed to ensure that major white matter bundles (e.g., corpus callosum, corticospinal tract) were reconstructed in accordance with anatomical expectations. The whole-brain mask was manually checked to ensure that only voxels within the brain parenchyma were used as seed points. Furthermore, a connection between two nodes was retained only if it was present in more than 50% of all subjects; otherwise, its connection strength was set to zero.

(3) Brain structural connectivity analysis

Brain structural connectivity, i.e., each connection preserved by the edges of the network (a 90 × 90 probability connectivity matrix) represents the strength of the probabilistic connections between the corresponding two brain regions. The general linear model (GLM) was used to statistically analyze each probability connection, and compare the intergroup differences between the patient group and the control group, controlling for the effects of gender, age, and education years. Threshold-free network-based statistics (TFNBS) were used for multiple comparison corrections. Threshold-free network-based statistics (TFNBS) was used for multiple comparison correction of structural connectivity differences. Specifically, 5000 permutations were performed to estimate the null distribution of the maximum cluster size. No pre-specified connection-level threshold was applied; instead, the TFNBS statistic was computed for all connections simultaneously. Cluster-level inference was conducted, and clusters with corrected *p*-values < 0.05 were considered statistically significant. The statistically significant connections after TFNBS correction were considered as having significant intergroup differences. The fiber connectivity probability between two regions was defined as the number of streamlines connecting them, normalized by the total number of streamlines originating from either region. The fiber connection probability was used as a measure of the strength of structural connectivity between these regions.

(4) Analysis of the topological properties of the brain structure network

Graph theoretical analysis was performed on weighted structural networks, where edge weights represented the row-normalized fiber connection probabilities between brain regions. To control for variations in network density, we calculated topological properties across a range of density thresholds from 0.05 to 0.5 (in increments of 0.01). The area under the curve (AUC) of each topological property across this density range was then computed and used as the final metric for statistical comparisons. Small-world metrics were calculated relative to 100 matched random networks generated for each subject, which preserved the same number of nodes, edges, and degree distribution as the actual network. Specifically: Gamma = Cp_actual/Cp_random (normalized clustering coefficient), Lambda = Lp_actual/Lp_random (normalized shortest path length), Sigma = Gamma/Lambda (small-worldness index). A network was considered to exhibit small-world properties when Sigma > 1.

The GRETNA software^2^ was used to calculate the topological attributes of the brain structure network, including global attributes, such as degree centrality (degree), global efficiency (gE), local efficiency (locE), clustering coefficients (Cp), shortest path length (Lp), normalized clustering coefficient (Gamma), normalized shortest path length (Lambda) and small-worldness (Sigma). Node attributes include degree centrality (Dc), betweenness centrality (Bc), and nodal efficiency (nodE).

General linear model was used to statistically analyze the global and node attributes, and compare the intergroup differences between the patient group and the control group, controlling for the effects of gender, age, and years of education. Global attributes were considered statistically significant at *p* < 0.05. The node attributes required corrections via FDR multiple comparisons. A *p*-value of <0.05 after FDR correction was considered statistically significant.

### 2.7 Statistical analysis

Clinical data of the two groups were statistically analyzed using SPSS software (version 19.0, IBM Corp., Armonk, NY, USA). The chi-square test was used for the inter-group comparisons to determine any gender differences. The two-sample *t*-test was used to compare the age and years of education between the two groups, and the GLM was used to compare the differences between the two groups on the score of the neuropsychological assessment scales while controlling for the effects of gender, age, and years of education, with *p* < 0.05 considered as being statistically significant. (The statistical analysis of brain structural connectivity and network topological properties were described previously). Partial correlation analysis of the brain image indices showing statistically significant inter-group differences was conducted with the scores of the neuropsychological assessment scale, while controlling for the effects of gender, age, and years of education. Partial correlation analyses were performed across all participants to examine the associations between the altered structural connections and cognitive performance. Bonferroni-corrected *p* < 0.05 was regarded as a significant correlation. In summary, age, gender, and years of education were included as covariates in all edge-level connectivity comparisons, graph-theoretical analyses and partial correlation analyses throughout this study.

## 3 Results

### 3.1 Demographic and clinical characteristics

The mean MoCA score of patients with MCI and the healthy controls was significantly different (*p* < 0.05). In contrast, the two groups did not differ significantly in terms of gender or years of education (*p* > 0.05; Table 1). Notably, the effects of age, gender, and education level were eliminated in the subsequent probabilistic fiber tracking, graph theory, and correlation analyses.

**TABLE 1:** Demographic and clinical characteristics of patients.

Variable	NC (*n* = 37)	WML-MCI (*n* = 33)	*P*-value
Gender (male/female)	20/17	16/17	0.642
Age/year	58.838 ± 7.801	64.000 ± 9.397	0.014*
Years of education/years	12.676 ± 2.839	11.545 ± 2.538	0.085
MoCA	27.703 ± 1.412	22.394 ± 2.499	<0.001*
Visual space and execution	4.595 ± 0.599	3.419 ± 1.177	<0.001*
Naming	2.973 ± 0.164	2.742 ± 0.631	0.077
Attention	5.892 ± 0.393	5.065 ± 1.153	0.002*
Language	2.459 ± 0.605	2.032 ± 0.547	0.003*
Abstraction	1.811 ± 0.569	1.387 ± 0.803	0.007*
Delayed recall	3.703 ± 0.996	1.613 ± 1.283	<0.001*
Orientation	6.000 ± 0.000	5.613 ± 0.715	0.017*

*Indicates a statistically significant difference between the two groups (*p* < 0.05).

The differences between the two groups were compared using the chi-square test for gender. The independent sample *t*-test was used to analyze age and years of education. The GLM was used for other scale scores, controlling for gender, age, and years of education. Statistical significance was set at *p* < 0.05. Furthermore, the radiological severity of white matter lesions (WMLs) in the WML-MCI group was characterized using the Fazekas scale. Among the 33 patients, 19 (57.6%) had a Fazekas score of 1, 10 (30.3%) had a score of 2, and 4 (12.1%) had a score of 3.

### 3.2 Probabilistic fiber tracking results

**FIGURE 1:**
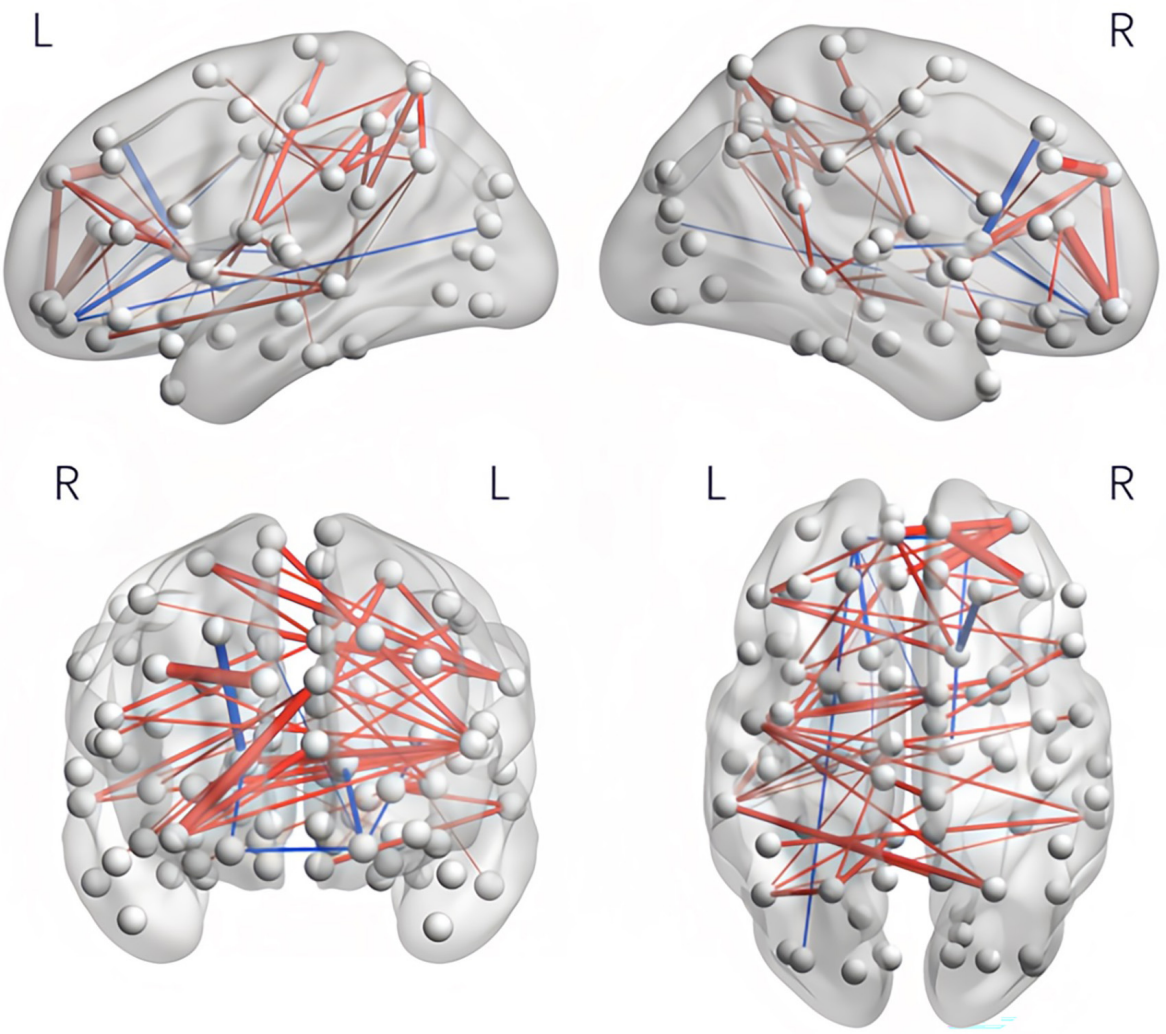
Results of the statistical analysis of fiber connection probability in the WML group and normal healthy group. The red-blue color represents the area with a statistically significant difference in fiber connection probability after the TFNBS multiple comparison correction (*p* < 0.05, after correction with TFNBS multiple comparison correction). Red represents elevated fiber connection probability, and blue represents decreased fiber connection probability.

A total of 2,939 connections (FWE-corrected, *p* < 0.05) were retained after the probabilistic fiber-tracking brain structural connectivity analysis. Of these, 50 connections showed statistically significant differences in probability values of fiber connectivity between the WML group and the healthy control group. Specifically, 42 fiber connections had increased probability values while 8 had decreased probability values (Figure 1).

Of the 42 connections with an increased probability of fiber connection, 30 (71.4%) were cortical-cortical connections, 13 were frontal-frontal connections, 6 were frontal-parietal connections, 3 were frontal-insular connections, 3 were frontal-temporal connections, and 2 were parietal-parietal connections. Fifteen were connected to the ipsilateral hemisphere and 15 to the bilateral hemisphere. There were 11 (26.2%) cortical deep gray matter (DGM) connections, mainly involving 6 frontal-basal ganglia connections and 1 (2.4%) DGM-DGM connection (Table 2).

**TABLE 2:** The pairs of brain regions with increased probability values of fiber connectivity.

WML > NC: 42				
No.	Row	Col	*T*-value	*P*-value
1	Left supramarginal gyrus	Right anterior central gyrus	2.722484	0.008
2	Right superior frontal gyrus, medial	Right middle frontal gyrus	4.191327	<0.001
3	Left insula	Right middle frontal gyrus	2.918665	0.005
4	Left inferior frontal gyrus, triangular part	Right middle frontal gyrus, orbital part	2.991787	0.004
5	Left superior frontal gyrus, medial	Right middle frontal gyrus, orbital part	3.747372	<0.001
6	Left anterior cingulate and paracingulate gyri	Right middle frontal gyrus, orbital part	3.433350	0.001
7	Right anterior cingulate and paracingulate gyri	Right middle frontal gyrus, orbital part	4.090178	<0.001
8	Left inferior frontal gyrus, triangular part	Right inferior frontal gyrus, opercular part	2.968762	0.004
9	Right medial and paracingulate gyri	Right inferior frontal gyrus, opercular part	3.064940	0.003
10	Left superior frontal gyrus, medial	Left inferior frontal gyrus, triangular part	3.061818	0.003
11	Right caudate nucleus	Left inferior frontal gyrus, triangular part	3.292993	0.002
12	Right anterior cingulate and paracingulate gyri	Right inferior frontal gyrus, orbital part	2.937227	0.005
13	Right superior parietal gyrus	Left Rolandic operculum	3.027037	0.004
14	Right paracentral lobule	Left Rolandic operculum	3.349012	0.001
15	Left lenticular putamen nucleus	Left Rolandic operculum	2.808564	0.007
16	Right lenticular putamen nucleus	Left Rolandic operculum	3.239468	0.002
17	Right lenticular globus pallidus	Left Rolandic operculum	3.119576	0.003
18	Right thalamus	Left Rolandic operculum	3.086957	0.003
19	Right middle temporal gyrus	Left Rolandic operculum	3.013993	0.004
20	Left medial and paracingulate gyri	Right Rolandic operculum	2.956798	0.004
21	Left supramarginal gyrus	Right supplementary motor area	2.937249	0.005
22	Left insula	Left superior frontal gyrus, medial	3.008920	0.004
23	Right insula	Left superior frontal gyrus, medial	2.926950	0.005
24	Right caudate nucleus	Left superior frontal gyrus, medial	3.214377	0.002
25	Left lenticular putamen nucleus	Left rectus gyrus	2.665118	0.010
26	Left middle temporal gyrus	Left rectus gyrus	3.155169	0.002
27	Left lenticular putamen nucleus	Left insula	3.472272	0.001
28	Left superior parietal gyrus	Left medial and paracingulate gyri	2.925846	0.005
29	Left supramarginal gyrus	Left medial and paracingulate gyri	2.804794	0.007
30	Left angular gyrus	Left medial and paracingulate gyri	2.832871	0.006
31	Left inferior temporal gyrus	Left medial and paracingulate gyri	2.687766	0.009
32	Left superior parietal gyrus	Left posterior cingulate gyrus	3.334076	0.001
33	Right thalamus	Left hippocampus	2.657700	0.010
34	Left angular gyrus	Right hippocampus	2.972940	0.004
35	Left lenticular putamen nucleus	Right amygdala	2.769773	0.007
36	Left angular gyrus	Left superior parietal gyrus	3.319638	0.001
37	Left supramarginal gyrus	Right superior parietal gyrus	3.476194	0.001
38	Right middle temporal gyrus	Left inferior parietal lobule	3.148106	0.002
39	Right precuneus	Left supramarginal gyrus	3.188370	0.002
40	Right middle temporal gyrus	Left angular gyrus	2.751608	0.008
41	Left middle temporal gyrus	Left lenticular putamen nucleus	3.068455	0.003
42	Right temporal pole: superior temporal gyrus	Right lenticular putamen nucleus	2.814732	0.006

The effects of gender, age, and years of education were controlled for, and the threshold-free network-based statistics (TFNBS) method was used to perform multiple comparisons between two brain regions with significant differences after corrections. All reported *p*-values are TFNBS-corrected.

Among the 8 connections with a reduced probability of fiber connections, 3 (37.5%) were cortical-cortical connections, mainly involving bilateral frontal lobes (2) and unilateral frontal-occipital lobes (1), and 3 (37.5%) were cortical-DGM connections, mainly involving frontal-basal ganglia nucleus (3 connections). The frontal lobe basal ganglia nucleus (3) was mainly involved, and 2 (25.0%) DGM-DGM connections, as shown in Table 3.

**TABLE 3:** The pairs of brain regions with a reduced probability of fiber connectivity.

LAMCI < NC: 8				
No.	Row	Col	*T*-value	*P*-value
1	Right caudate nucleus	Right dorsolateral superior frontal gyrus	−3.608215	0.001
2	Right superior frontal gyrus, orbital part	Left superior frontal gyrus, orbital part	−2.951039	0.004
3	Right medial and paracingulate gyri	Left superior frontal gyrus, orbital part	−2.696953	0.009
4	Left middle occipital gyrus	Left superior frontal gyrus, orbital part	−2.892704	0.005
5	Left caudate nucleus	Left superior frontal gyrus, orbital part	−3.159109	0.002
6	Right caudate nucleus	Right superior frontal gyrus, orbital part	−2.885020	0.005
7	Left thalamus	Left caudate nucleus	−2.678062	0.009
8	Right thalamus	Right caudate nucleus	−2.975723	0.004

The effects of gender, age, and years of education were controlled for. The threshold-free network-based statistics (TFNBS) was used to perform multiple comparisons between two brain regions with significant differences after corrections. All reported *p*-values are TFNBS-corrected.

### 3.3 Results of graph theory analysis

Global attributes include connection strength (degree), global efficiency (gE), and local efficiency (locE). No significant statistical differences were found in the shortest path length (Lp), clustering coefficient (Cp), and small worldness attributes (Lambda, Gamma, Sigma) (Table 4).

**TABLE 4:** Results of graph theory analysis between the two groups (mean ± SD), F statistics were derived from the GLM analysis controlling for age, sex, and education.

Network metric	WML group (*n* = 33)	NC group (*n* = 37)	*F*-value	*P*-value
Degree	0.510 ± 0.061	0.501 ± 0.035	1.248	0.268
gE	0.027 ± 0.003	0.026 ± 0.002	1.495	0.226
locE	0.028 ± 0.003	0.027 ± 0.002	2.449	0.123
Lp	37.697 ± 4.284	38.092 ± 2.616	0.660	0.420
Cp	0.003 ± 0.000	0.003 ± 0.001	2.666	0.108
Lambda	1.172 ± 0.016	1.176 ± 0.016	0.927	0.339
Gamma	1.587 ± 0.215	1.593 ± 0.163	0.121	0.730
Sigma	1.353 ± 0.164	1.354 ± 0.124	0.232	0.631

The GLM analysis revealed no significant differences between the two groups after controlling for age, gender, and education. Similarly, there were no significant differences in the nodal degree, nodal betweenness, nodal gE, nodal locE, nodal Lp, and nodal Cp of the node attributes.

### 3.4 The relationship between abnormal structural connectivity and cognitive function

After controlling for gender, age, and years of education, the probability values of the fiber connections and the MOCA scores from significant inter-group differences were analyzed by partial correlation analysis. Partial correlation analyses were performed across all participants to examine the associations between the altered structural connections and cognitive performance.

Bonferroni correction was applied to account for multiple comparisons. Based on the 50 statistical tests performed, the significance threshold was adjusted to 0.05/50 = 0.001.

The results revealed that eight altered connections (comprising 5 cortico-cortical and three cortico-deep gray matter connections) exhibited significant negative correlations with specific cognitive domains and the total MoCA score (Table 5).

**TABLE 5:** Correlation analysis of fiber connection probability values between two brain regions with significant inter-group differences in neuropsychological assessment.

Pairs of brain regions		Cognitive scales	*P*-value	*r*-value
Right lenticular globus pallidus	Left Rolandic operculum	Language	<0.001	−0.434*
Right middle temporal gyrus	Left Rolandic operculum	Attention	<0.001	−0.453*
Right insula	Left superior frontal gyrus, medial	Visual space and execution	<0.001	−0.431*
Left lenticular putamen nucleus	Left insula	MoCA total score	<0.001	−0.423*
		Delayed recall	<0.001	−0.432*
Left superior parietal gyrus	Left medial and paracingulate gyri	MoCA total score	<0.001	−0.447*
		Delayed recall	<0.001	−0.428*
Left supramarginal gyrus	Right superior parietal gyrus	MoCA total score	<0.001	−0.417*
		Visual space and execution	<0.001	−0.442*
Right precuneus	Left supramarginal gyrus	MoCA total score	<0.001	−0.462*
		Visual space and execution	<0.001	−0.462*
		Abstraction	<0.001	−0.457*
Left middle temporal gyrus	Left lenticular putamen nucleus	MoCA total score	<0.001	−0.407*
		Delayed recall	<0.001	−0.419*

Partial correlation analysis was conducted on the fiber connection probability values of two brain regions with significant inter-group differences and the scores of neuropsychological assessment scales, controlling for the effects of age, sex, and education. Bonferroni correction was applied for 50 comparisons. The significance threshold was set at α = 0.05/50 = 0.001. The *p*-values shown in the table are uncorrected. An asterisk (*) indicates uncorrected *p* < 0.001, i.e., the correlation remained significant after Bonferroni correction.

## 4 Discussion

This study employed an integrated approach combining whole-brain probabilistic fiber tracking with graph theoretical analysis to investigate alterations in the structural connectome of patients with WML-related MCI. The principal findings are threefold. First, at the connectional level, patients exhibited a complex pattern of both decreased and increased structural connectivity probabilities. Second, despite these localized alterations, the global topological properties of the brain network, as assessed by graph theory metrics, remained preserved. Third, the strength of several altered connections was significantly correlated with cognitive performance, underscoring their potential clinical relevance. These findings indicate that the early stage of cognitive impairment associated with white matter lesions (WML) is characterized by dynamic reorganization of the structural connectome, wherein focal disconnections coexist with widespread increases in structural connectivity, while global network efficiency remains preserved. This pattern of “local disruption–widespread compensation–global preservation” may reflect an adaptive regulatory mechanism engaged by the brain network in response to white matter damage, reflecting intrinsic neuroplasticity and providing new insights into the neurobiological underpinnings of WML-related cognitive decline.

White matter lesions are a common cause of vascular cognitive impairment (Biesbroek et al., 2017; Lee et al., 2025). Many studies have confirmed the correlation between the imaging features of WMLs and cognitive impairment (Wang et al., 2022). As the physical connections between gray matter regions, the integrity of white matter fibers is crucial for efficient interregional communication. WMLs can induce axonal loss and gliosis, thereby altering the diffusion properties of water molecules in neural tissue and ultimately manifesting as changes in diffusion tensor imaging (DTI) parameters (Lin et al., 2015). For this reason, DTI offers a unique *in vivo* perspective on the microstructural integrity and damage of white matter, holding promise as a potential biomarker for disease diagnosis and severity assessment.

This study demonstrates that whole-brain connectome analysis based on probabilistic fiber tracking is an effective approach for uncovering structural abnormalities underlying cognitive decline associated with white matter lesions (WMLs) (Bastiani et al., 2017; Chen et al., 2023).

To minimize the risk of false-positive connections inherent to probabilistic tractography, a stringent connection retention criterion was applied–only inter-regional connections consistently present in at least 50% of participants were included. Without imposing any *a priori* assumptions, whole-brain analysis revealed that structural connectivity alterations in WML-related cognitive impairment are primarily concentrated in two types of pathways: (1) cortico–cortical connections between bilateral frontal lobe regions, and (2) cortico–subcortical (deep gray matter) connections between the frontal lobe and basal ganglia. These findings further underscore the central role of the prefrontal cortical network and its structural connections with the basal ganglia in supporting cognitive functions (Vossel et al., 2014; Menon and D’Esposito, 2022). Disruption of these critical white matter pathways may lead to disconnection within cortico–cortical or cortico–subcortical circuits, thereby impairing specific cognitive domains such as executive function and attention. Notably, similar patterns of structural network reorganization have been reported in other neurodegenerative disorders, suggesting a potential transdiagnostic pathological mechanism (Mishra et al., 2020). Moreover, this study identified significant connectivity abnormalities involving the parietal cortex at the early stage of cognitive impairment–a finding consistently observed in other neurocognitive disorders –indicating that the parietal cortex may represent a common early-vulnerable region across diverse etiologies of cognitive decline.

Notably, patients with WML-related mild cognitive impairment (WML-MCI) exhibited a substantially greater number of connections with significantly increased connectivity probability compared to those with decreased connectivity (42 increased vs. 8 decreased). These enhanced connections were primarily located in cortico–cortical pathways between the frontal lobe and other cortical regions. This striking and somewhat counterintuitive finding suggests that the brain’s structural network may undergo widespread compensatory reorganization in response to early white matter damage. A plausible explanation is that the brain strengthens alternative or redundant pathways to bypass disrupted core circuits and thereby maintain cognitive function. Such upregulation of structural connectivity may represent a form of neuroplasticity, analogous to the adaptive remodeling mechanisms observed in other neurological disorders. Nevertheless, the observed “connectivity enhancement” should be interpreted with caution. Previous studies have proposed several plausible explanations: such increases may partly stem from inherent methodological limitations of diffusion MRI –based probabilistic tractography. For instance, pathological processes such as demyelination alter the diffusion properties of water molecules, which can lead probabilistic tractography algorithms to overestimate actual structural connectivity strength. Moreover, these apparent “enhancements” may not reflect active, beneficial neurocompensatory mechanisms, but rather represent epiphenomena or secondary consequences associated with underlying white matter pathology.

While compensatory reorganization represents a plausible explanation, the observed increase in connectivity derived from probabilistic tractography may also be influenced by methodological factors, such as altered diffusion properties, registration or ROI-related effects, and false-positive streamlines. Therefore, these findings should be interpreted as potential evidence of compensation rather than a definitive conclusion. To clarify their true biological nature, future research should integrate advanced diffusion imaging techniques with histopathological validation. Overall, the co-occurrence of both decreased and increased connections reveals a highly heterogeneous and dynamically evolving pattern of structural network alterations in the early stages of cognitive impairment.

The analysis of structural connection groups through graph theory can provide useful network organization information (Insabato et al., 2019). In our study, the graph analysis of global network attributes did not demonstrate any significant differences. A longitudinal study of cerebral small vessel disease (SVD) by Tuladhar et al. (2020) found that a decline in global efficiency of the white matter structural brain networks in patients with mild to moderate white matter damage was not significant, indicating that although behavioral changes or functional connectivity abnormalities can occur at this stage, the anatomic connectivity is relatively preserved. These results suggest that in terms of the basis of functional expression, the structural connectivity network has a more stable and effective organizational pattern.

This study has some limitations. First, although the WML-MCI group and healthy controls were matched as closely as possible, the WML-MCI group was significantly older than the control group. While our statistical models rigorously controlled for age, sex, and education as covariates, we acknowledge that residual age-related confounding cannot be completely excluded. Given the profound influence of aging on white matter integrity, WML burden, and diffusion MRI metrics, this imbalance may still exert subtle effects on the reported connectivity differences. Therefore, the interpretation of our findings should consider this potential demographic bias. Second, it is a cross-sectional study. Longitudinal studies are needed to evaluate the dynamic changes in brain structural networks and cognitive functions. Third, the sample size was relatively small, and the relationship between white matter lesion (WML) burden severity and structural connectivity alterations remains unresolved. Although Fazekas scores were reported, WML burden was heavily skewed toward mild-to-moderate cases (87.9%), with only 4 patients (12.1%) having severe WMLs. Therefore, we cannot determine whether the increased or decreased connectivity probabilities are proportional to WML severity or reflect a threshold effect. Future longitudinal studies with larger, more balanced samples across the full severity spectrum are needed. Furthermore, as the Fazekas scale is ordinal, future studies using quantitative volumetric measurements of WMLs may provide more sensitive assessments of lesion burden. Fourth, another limitation concerns the interpretation of the brain–behavior correlation analyses. Because the correlations were conducted using connections that had already demonstrated significant between-group differences, there is a potential risk of selection bias or circularity. Moreover, the correlations were performed across all participants rather than within the WML-MCI group alone; therefore, some associations may partly reflect overall group separation instead of continuous brain–behavior relationships within patients. Consequently, these findings should be considered exploratory and interpreted with caution. Future studies with larger cohorts and independent validation analyses are needed to confirm the robustness of these associations. Finally, the present study lacked a control group of MCI patients without white matter lesions. Consequently, we cannot fully dissociate the effects of WMLs from those of MCI itself on the observed structural network alterations. Future studies should include such a comparison group to determine the specific contribution of WMLs to connectome reorganization in the context of MCI.

## 5 Conclusion

By integrating probabilistic fiber tracking with whole-brain structural connectome analysis based on graph theory, this study identifies specific alterations in cortico–cortical and cortico–deep gray matter connectivity associated with cognitive decline in patients with white matter lesion (WML)-related cognitive impairment. These structural connectivity disruptions may represent a potential pathological substrate of cognitive dysfunction. While these findings provide valuable insights, further longitudinal studies and external validation are required to determine their utility as diagnostic or prognostic tools.

## Data Availability

The raw data supporting the conclusions of this article will be made available by the authors, without undue reservation.

## Footnotes

1
http://www.nitrc.org/projects/panda/

2
https://www.nitrc.org/projects/gretna/
